# Prey-tracking behavior and prey preferences in a tree-climbing firefly

**DOI:** 10.7717/peerj.8080

**Published:** 2019-12-16

**Authors:** Nozomu Sato

**Affiliations:** 1 Faculty of Agriculture and Life Science, Hirosaki University, Hirosaki, Aomori, Japan; 2 Current affiliation: Graduate School of Urban Environmental Sciences, Tokyo Metropolitan University, Hachiouji, Japan

**Keywords:** Arboreal, Predator-prey interaction, *Pyrocoelia atripennis*, Operculum, Lampyridae, Land snail, Mucus, Bradybaenidae, Cyclophoridae

## Abstract

Prey-tracking behavior is common in snail-killing predators, but in the family Lampyridae, this behavior has been validated in only a single species even though this Coleopteran family includes many specialist snail predators. The endemic firefly *Pyrocoelia atripennis* is a major snail-killing predator in the Yaeyama Islands of Japan, and the larvae often climb on the trees and grasses at night. This tree-climbing behavior is relevant to larval food choices and anti-predatory defenses of land snails. This study examined whether lampyrid larvae can track snail mucus trails and examined larval prey preferences using alternative choice experiments. In addition, predation trials were conducted to evaluate which snail species are potential prey. *P. atripennis* larvae significantly selected mucous trails over distilled water or control (no-trail) treatments. In addition, a semi-arboreal species was preferred over a ground-dwelling species. In predation trials, the larvae preyed on five out of 10 endemic snail species, all of which were semi-arboreal or arboreal species. Ground-dwelling Cyclophoridae and *Aegista* species have effective anti-predatory defenses consisting of an operculum or “foamy-lid” that fills the shell aperture. Whether the prey has a lid affects the predation success of lampyrid larvae, and larval tree-climbing behavior may be an adaptation used to search for semi-arboreal and arboreal land snails that lack defensive lids. Furthermore, snail mucus left on the plant stem may help the lampyrid larvae to locate their prey.

## Introduction

Carnivorous predators often use signs of the previous presence of potential prey, such as excrement, food remains, footprints, odors, pheromones, and nests to locate their food.

Some of them have a strategy for finding prey by tracing olfactory or visual signals that remain after prey moves. This strategy, called “prey-tracking behavior” has been observed in vertebrates and invertebrates (Furry, Swain & Chiszar, 1991; Mc Donnell, Paine & Gormally, 2007; Iwai, Sugiura & Chiba, 2010; Lai, Chen & Lee, 2011; Holland et al., 2012). During locomotion, terrestrial gastropods leave a distinct trail of mucus which consists of water combined with mucin or mucin-like carbohydrate-protein complexes and some sexual pheromones (Davies & Hawkins, 1998; Holland, Gousy Leblanc & Yew, 2018). These trails are used in a wide range of intraspecific social interactions (Ng et al., 2013). Moreover, some studies suggest that snail-eating predators may detect chemical cues from the mucous trails and tracking to locate prey. The land flatworm *Platydemus manokwari* (Tricladida: Rhynchodemidae) and the rosy wolf snail *Euglandina rosea* (Pulmonata: Spiraxidae) are invasive snail-killing predators of land snails in the Pacific islands. These predators track mucous trails and prey on snails both on the ground and in trees (Iwai, Sugiura & Chiba, 2010; Sugiura & Yamaura, 2010; Holland et al., 2012). They have thereby caused catastrophic losses among all island-specific ground-dwelling and arboreal snail communities (Cowie, 2001; Sugiura, 2009; Iwai, Sugiura & Chiba, 2010; Holland et al., 2012). Thus, prey-tracking behaviors have been observed in these snail-killing specialist predators. However, there is little evidence regarding snail-feeding insects (Mc Donnell, Paine & Gormally, 2007; Digweed, 1994). In the Lampyridae (Insecta: Coleoptera), this behavior was first recorded in a European firefly *Lampyris noctiluca* (Schwalb, 1961). However, there are no reports of this behavior in other snail-eating firefly species.

The genus *Pyrocoelia* (Lampyridae) is mainly distributed from the tropics to temperate regions in East Asia, including the continental islands of the Pacific Ocean. In the larval stage, all *Pyrocoelia* species are specialist predators on land snails. *P. pectoralis*, a large species native to in China, reaches 50 mm or more in the last (6th) instar larvae and preys on large land snails. The larval period is 1 to 2 years or more, and larvae of different ages and sizes co-occur in the environment. Therefore, they potentially function as a biological agent for controlling pest land snails (Fu & Meyer-Rochow, 2013). The larval predation method was described by Wang et al. (2007) and Lewis (2016). When a lampyrid larva encounters a snail, (i) the larva bites one of the snail’s anterior tentacles with its mandible and injects digestive juice (midgut extracts) into the snail body, (ii) the snail stops moving and withdraws into its shell, and the larva climbs onto the shell, (iii) the larva repeatedly bites one of the anterior tentacles while remaining attached to the shell with a sucker-like organ (pygopodium) located on the terminal segment of the abdomen, and (iv) the larva consumes the snail after it has been paralyzed by the toxin (Copeland, 1981) and has completely ceased movement.

Another feature of *Pyrocoelia* species is that they climb the grasses or trees after sunset, returning to the ground before sunrise (Wang et al., 2007). This tree-climbing behavior has been reported in many genera of lampyrid around the world: including *Alecton* (Madruga Rios & Hernández Quinta, 2010), *Aspisoma* (Viviani, Rosa & Martins, 2012), *Lamprigera*, *Lamprocera* (Viviani, 2001), *Lampyris* (Schwalb, 1961; De Cock, 2009), *Lychnuris*, *Nyctophila*, and *Pyractomena* (Buschman, 1984; Day, 2011). In Japan, however, it is known only in *Pyrocoelia*. In particular, it is often observed in an endemic firefly *Pyrocoelia atripennis* inhabiting the Yaeyama archipelago, Okinawa, Japan. The author has observed that *P. atripennis* larvae prey on arboreal and semi-arboreal land snails on trees at night. Therefore, tree-climbing behavior is likely a larval feeding strategy to locate land snails on plants. This raises the question of why the larvae would expend additional time and energy to climb trees to eat arboreal snails when many snails occur on the ground.

On the Yaeyama Islands, the land snail family of Cyclophoridae is dominant on the ground surface, and all of them have an operculum (shell lid). The operculum is a corneous or calcareous tectiform structure that closes the shell aperture tightly like a trapdoor to protect the body from desiccation and predation (Kelly & Cory, 1987; Norton, 1988). As lamprid larvae are predators that invade through the shell aperture, land snails with an operculum can be difficult prey. Therefore, the phylogenetically inoperculate group of land snails (e.g., Bradybaenidae and Camaenidae) should be easier prey for the larvae. Although these snails try to defend by emitting mucous bubbles in their shell aperture when attacked by predators, this has a limited protective effect against fireflies (Madruga Rios & Hernández Quinta, 2010). Many of these land snails are also found on plants. Therefore, the hypothesis in this study is that the tree-climbing behavior of *P. atripennis* is a feeding strategy adapted for preying on inoperculate land snails living on plants while avoiding operculate species.

Previous studies have not revealed the relationship between the feeding preferences of lampyrid larvae and tree-climbing behavior. Accordingly, this article first examined whether *P. atripennis* larvae demonstrate prey-tracking abilities. Second, the author tested whether the tree-climbing larvae prefer land snails that live on plants to those on the ground by using land snails of three habitat types (arboreal, semi-arboreal, ground-dwelling). The larvae were expected to prefer the mucus of arboreal and semi-arboreal snails to that of ground-dwelling snails. Finally, this study investigated the antipredator defenses of several species of terrestrial snails against predation by lampyrid larvae. If the larva can track mucous trails and distinguish species by their mucus, and if prey preference is related to the defense ability and habitat of the prey species, larval tree-climbing behavior would be an adaptive foraging behavior.

## Materials and Methods

### Study species

**Figure 1:**
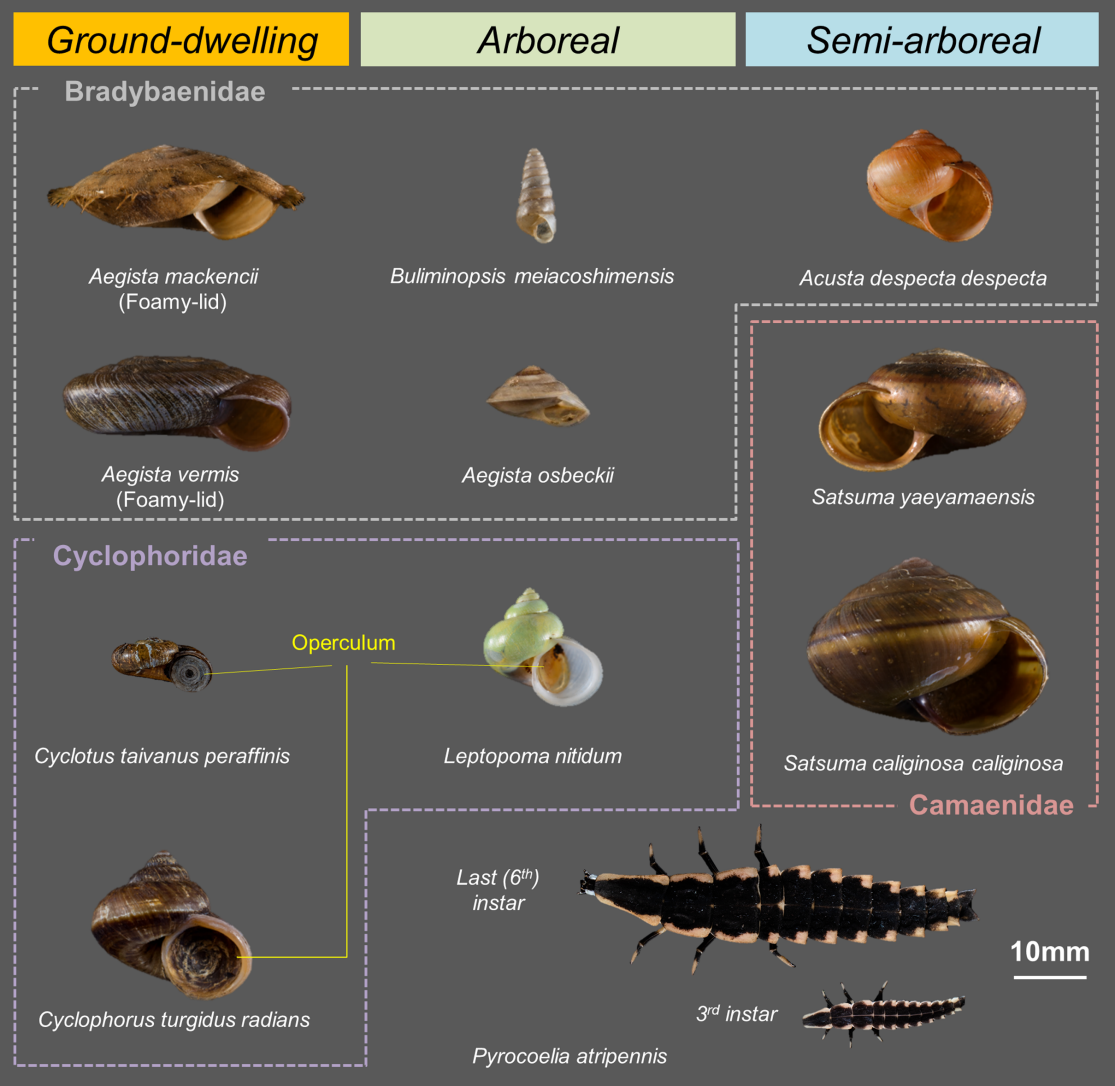
Land snails and lampyrid larvae used in this study. All species inhabit Ishigaki Island, Okinawa, Japan. Snail species in the first column are ground-dwelling, in the second column arboreal and in the last column, semi-arboreal. Species within the same dashed line are members of the same family. All species are shown to the same scale.

Lampyrid larvae and land snails were collected for laboratory experiments in mid-October 2017 on Ishigaki Island, Okinawa, Japan. This island lies in the Yaeyama archipelago (24°00′–24°40′N and 122°45′–124°30′E), which includes Iriomote Island and 21 other small islands. These islands are characterized by subtropical evergreen broad-leaved forest. The annual rainfall is 2,106.8 mm, and the annual mean temperature is 24.3 °C. Land snails and lampyrid larvae are active almost all year. On five islands of Yaeyama (Iriomote, Ishigaki, Taketomi, Yufu, Kohama), the endemic firefly *P. atripennis* is common and inhabits the area from the forest floor to the grassland at the forest edge, along with land snails. Larvae are larger than all the endemic land snail species and are therefore potentially successful predators on them. Therefore, 10 common endemic land snails that inhabit the same environment as the fireflies were collected (Fig. 1): *Acusta despecta despecta*, *Aegista mackensii*, *Aegista osbeckii*, *Aegista vermis*, *Buliminopsis meiacoshimensi*, *Cyclophorus turgidus radians*, *Cyclotus taivanus peraffinis*, *Leptopoma nitidum*, *Satsuma caliginosa caliginosa*, and *Satsuma yaeyamaensis*. All specimens were kept under laboratory conditions at 24 °C with a 13:11 light/dark photoperiod. All experiments were conducted from early November 2017 to mid-January 2018.

### Choice experiments: mucus trail-tracking and prey preference

**Figure 2:**
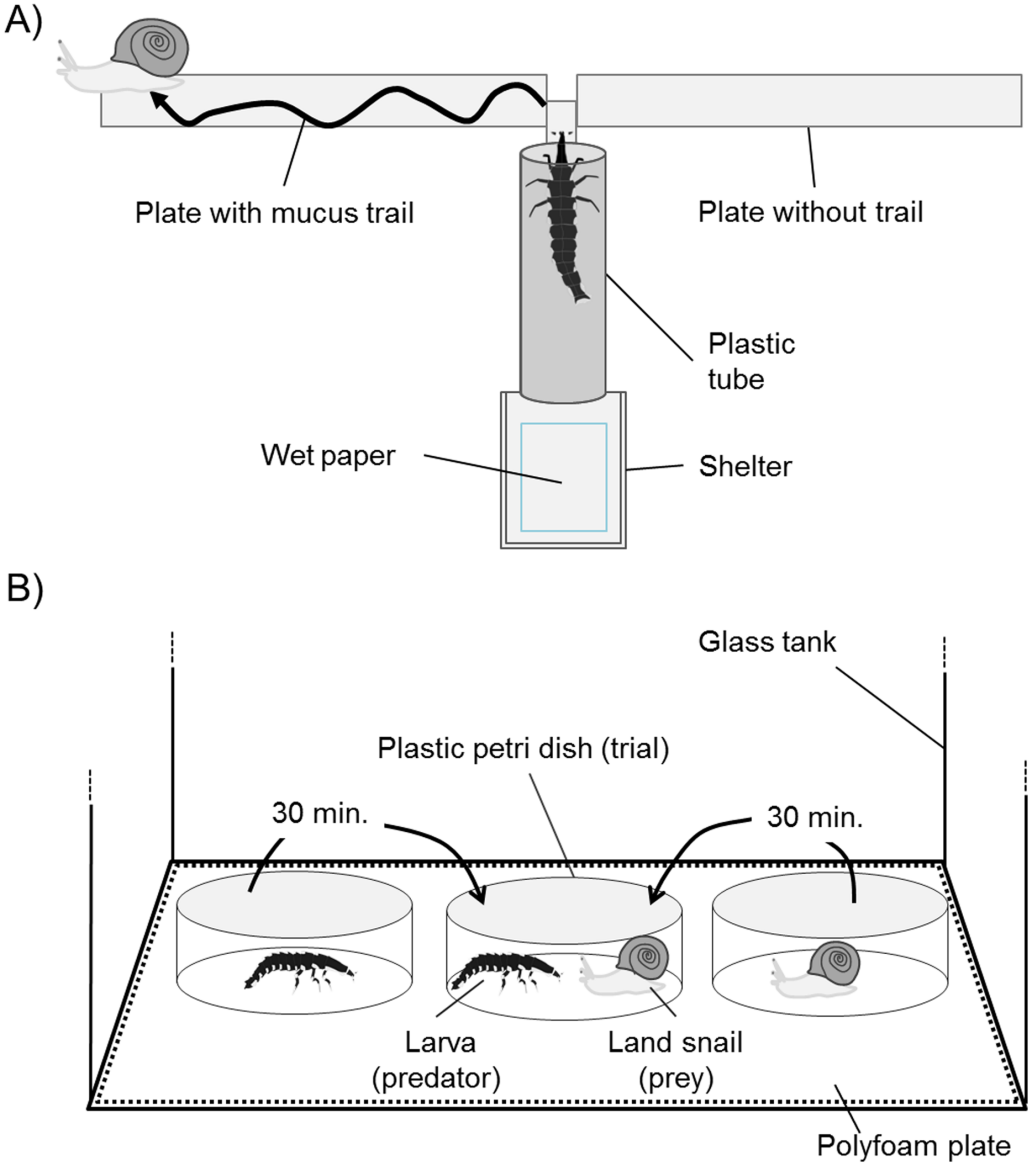
Schematic representation of apparatus for mucus trail-tracking experiments viewed from above (A) and for predation trials viewed from the side (B).

To evaluate the tracking ability of *P. atripennis* larvae, several alternatives were provided in Experiment 1: no-trail (control) vs. distilled water (DW), mucous trail vs. no trail, and mucous trail vs. DW, 15 iterations each. The prey-tracking experiment design was based on those of Iwai, Sugiura & Chiba (2010) and Holland et al. (2012). For the present study, an experimental apparatus was created using a shelter combined with wood plates (right and left the side, 100 × 10 mm), plastic tubing (12 mm in diameter, 60 mm long), and another wood plate (central plate) under the tube (Fig. 2A). The shelter and its bottom, which served as the larval starting point, were covered by cotton wool moistened with DW. The tip of the central plate extended (10 × 3 mm) from the tube entrance and bisected the two side plates at half their width (i.e., created a 5 × 3 mm gap). This experimental apparatus was set inside a glass aquarium (350 × 210 × 240 mm) with a wet paper towel on the bottom and was kept at 24 °C and 70–80% humidity with a 13:11 light/dark photoperiod (the field conditions under which larvae are active). The entire apparatus was set at a height of 12 cm from the bottom of the aquarium.

The mucus used was from *S. yaeyamaensis* (shell width mean ± SD: 22.7 ± 1.2 mm, weight: 2.47 ± 0.26, three individuals), which has been confirmed as prey of *P. atripennis*. Mucous trails were made on wood plates by placing a snail at the middle of the plate’s edge and sliding them across to the other edge of the plate. DW trails were painted using a brush so as to be equal to the width of snail mucus trails. The direction of the trail was carefully chosen to run outward from the junction of the side plates and the center plate, and the right and left positions were set randomly. Mucous trails were used within 2 h because studies on other mucus-tracking species have shown that responsiveness to the trail decreases as the mucus dries (Mc Donnell, Paine & Gormally, 2007). The experiment was conducted in the dark (a red LED light was used only when preparing for the experiment) because both lampyrid larvae and snails are nocturnal and extremely sensitive to artificial light. The larvae were randomly selected from among the individuals performing the target activity (walking and glowing) within the breeding container 30 min after turning off the lights. Larval body size and weight were 30.2 ± 8.0 mm and 0.20 ± 0.21 g (*n* = 45) in this experiment. Larvae remained still or moved around in the shelter for a while before passing through the tube and emerging on the plate, where they reached the left and right trail plates.

Larval choice was defined as the first plate occupied by the entire body. The experiment was recorded using an infrared camera (SONY HDR-CX 560) from before the larva exited the tube entrance until it came back to the center after walking on both plates. The time (s) spent by the larva on each trail before returning to the center was recorded (staying time). Larvae appeared to intensively examine an area in which they detected mucus, so it is expected that the staying time will be longer in the trail with mucus. The plates used in the experiments were washed well with alkaline detergent and DW, then dried completely, before being used again for another trial.

Experiment 2 examined whether the lampyrid larvae distinguished snail species by the mucous trail and assessed their feeding preference. The experiment used three snail species with different habitat types: *A. mackensii* (ground-dwelling), *Leptopoma nitidum* (arboreal), and *S. c. caliginosa* (semi-arboreal). Their shell widths and weights were 24.0 ± 2.8 mm and 1.94 ± 0.61 g, 11.6 ± 0.5 mm and 0.45 ± 0.03 g, and 25.1 ± 1.2 mm and 2.84 ± 0.96 g (*n* = 3 for each species), respectively. The habitat types of the land snails were identified using a local red list for Okinawa Prefecture (2017) and direct observations in the field. The width of the mucous trail was about the same between the left and right plates. In Experiment 2, larval choice frequency and staying time were measured using the following three combinations: ground-dwelling vs. arboreal, ground-dwelling vs. semi-arboreal, and arboreal vs. semi-arboreal, 15 replicates each. The body size of the firefly larvae was 29.3 ± 8.6 mm, and the bodyweight was 0.16 ± 0.20 g; 45 individuals were used in this experiment. Other methods conformed to those in Experiment 1.

### Predation trials

I investigated whether larvae preyed on 10 species of land snails presumed to be potential prey species of *P. atripennis* in the Ishigaki islands: *Acusta despecta despecta* (*n* = 9), *A. mackensii* (*n* = 4), *Aegista osbeckii* (*n* = 5), *A. vermis* (*n* = 5), *Buliminopsis meiacoshimensi* (*n* = 3), *Cyclophorus turgidus radians* (*n* = 5), *Cyclotus taivanus peraffinis* (*n* = 3), *Leptopoma nitidum* (*n* = 3), *S. c. caliginosa* (*n* = 4), and *S. yaeyamaensis* (*n* = 5).

The larval body size of *P. attripennis* was 31.9 ± 10.6 mm and the body weight was 0.29 ± 0.28 g, and 21 individuals were used in the predation trials. The same snail species was not given twice to one larva. The predation trials were conducted in a glass tank (315 × 315 × 480 mm, L × W × H) with a polyfoam plate on the bottom in the dark at 24 °C and >80% humidity (Fig. 2B). The trial was recorded from above with an infrared video camera. First, a land snail and a lampyrid larva were placed in separate plastic petri dishes (100 mm diameter, 40 mm height) in the tank. After a 30 min habituation period, the snail and larva were carefully placed in the same petri dish so as not to disturb them by hand. A determination was made as to whether predation was possible by observing whether the larvae showed predatory behavior (climbing on the shell or biting the snail head) until predation succeeded (i.e., the snail ceased to move) or 15 min had elapsed after the larvae had stopped attempting predation. All larval body sizes were larger than the snail shell diameters in each trial because arthropod and other snail-feeding predators generally prefer prey smaller than their body size (Lai, Chen & Lee, 2011; Remmel, Davison & Tammaru, 2011; Holland et al., 2012).

No insects or land snails used in this study were protected species in Japan. All experiments and trials were undertaken according to the Hirosaki University Animal Experimentation Regulations and comply with the current laws of Japan.

### Statistical analysis

The frequencies of the trail that the larva initially selected in Experiments 1 and 2 were analyzed using a two-tailed binomial test, and exact binomial 95% confidence intervals (CI) were calculated. A two-tailed paired *t*-test was used to compare the staying time on both trails for Experiments 1 and 2. The data on larvae that fell off the plate along the way would affect the staying time analysis and were excluded. When the larvae occupied only one plate and returned to the inside of the tube, the staying time on the opposite plate was set to zero. All analyses were performed with R ver. 3.4.4 (R Development Core Team, 2018).

## Results

### Mucus trail-tracking

**Figure 3:**
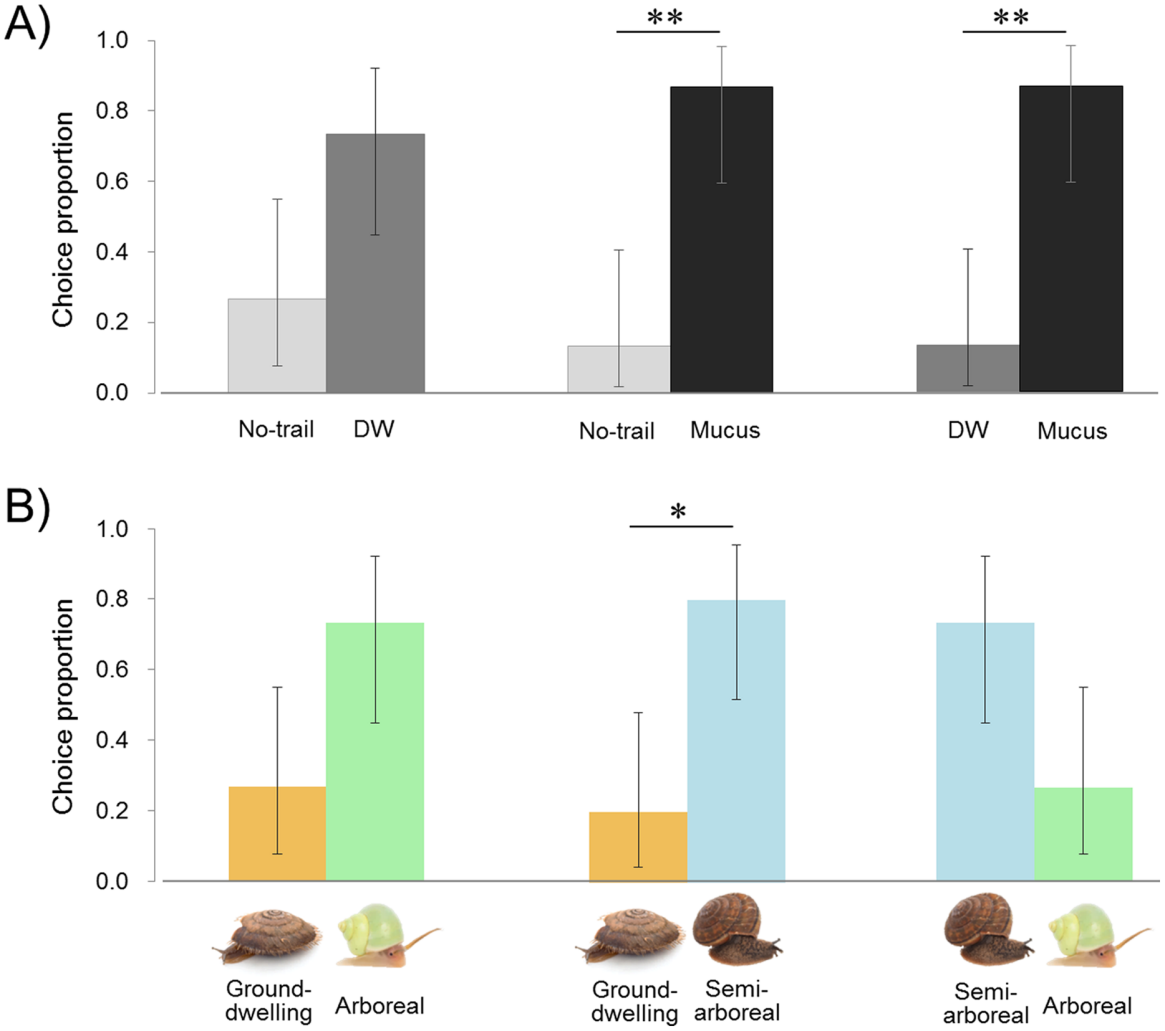
Results of trail-tracking experiments with *Pyrocoelia atripennis* larvae. (A) Proportion of individuals selecting no trail vs. distilled water (DW), no trail vs. snail mucus trail, DW vs. snail mucus trail. (B) Proportion of individuals selecting mucus trails from three species of snails with different habitat types: *Aegista mackensii* (ground-dwelling) vs. *Satsuma caliginosa caliginosa* (semi-arboreal) vs. *Leptopoma nitidum* (arboreal). *n* = 15 for all comparisons.The error bar shows 95% exact binomial confidence intervals (CI). Asterisk indicates *P* < 0.05 (*) and *P* < 0.01 (**).

Lampyrid larvae tended to choose the DW plate with moisture on the surface rather than no-trail, and also to distinguish mucus from controls (DW or no-trail). The larvae moved their heads from side to side, stopped when touching the mucus on the plate, and moved slowly as if assessing the smell of mucus (Movie S1). They followed the snail mucus and reached the end of the trail, and then returned the way they came. The larvae tended to choose the DW plate more often than the no-trail plate (11 of 15 replicates, exact binomial (95% CI [44.9–92.2 and 7.8–55.1]), *P* = 0.1185) (Fig. 3A; Table S1). The plate with the snail mucus trail was chosen by larvae over DW and no trail (13 of 15 replicates in both cases (95% CI [59.5–98.3 and 1.7–40.5]), *P* = 0.0074). Comparison of the larval staying time on both plates on each trial shows that larvae stayed significantly longer on the mucus than on the DW trail, 123 ± 66 and 43 ± 32 s (two-tailed paired *t*-test, *t* = −3.430, df = 12, *P* = 0.0049) and no trail, 95 ± 62 and 34 ± 25 s (*t* = −3.359, df = 13, *P* = 0.0051). The staying time tended to be longer with DW than no trail, 96 ± 74 and 67 ± 35 s (*t* = −1.941, df = 14, *P* = 0.0726).

### Prey preference

The arboreal *L. nitidum* tended to be chosen more than the ground-dwelling *A. mackensii* (11 of 15 replicates, exact binomial (95% CI [44.9–92.2 and 7.8–55.1]), *P* = 0.1185) (Fig. 3B; Table S1). The semi-arboreal *S. c. caliginosa* tended to be chosen more than arboreal *L. nitidum* (11 of 15 replicates (95% CI [44.9–92.2 and 7.8–55.1]), *P* = 0.1185) and more frequently than ground-dwelling *A. mackensii* (12 of 15 replicates (95% CI [51.9–95.7 and 4.3–48.1]), *P* = 0.0352). Larvae stayed longer on the semi-arboreal than on the arboreal snail trail, 172 ± 127 and 101± 52 s (two-tailed paired *t*-test, *t* = 2.263, df = 14, *P* = 0.0400). However, the staying time on the semi-arboreal snail trail did not differ from that on the ground-dwelling snail trail, 140 ± 99 and 132 ± 90 s (*t* = 0.247, df = 13, *P* = 0.8090). Also, the staying time on the arboreal snail trail did not differ from that on the ground-dwelling snail trail, 128 ± 57 and 98 ± 62 s (*t* = − 1.371, df = 14, *P* = 0.1918).

### Predation trials

**Figure 4:**
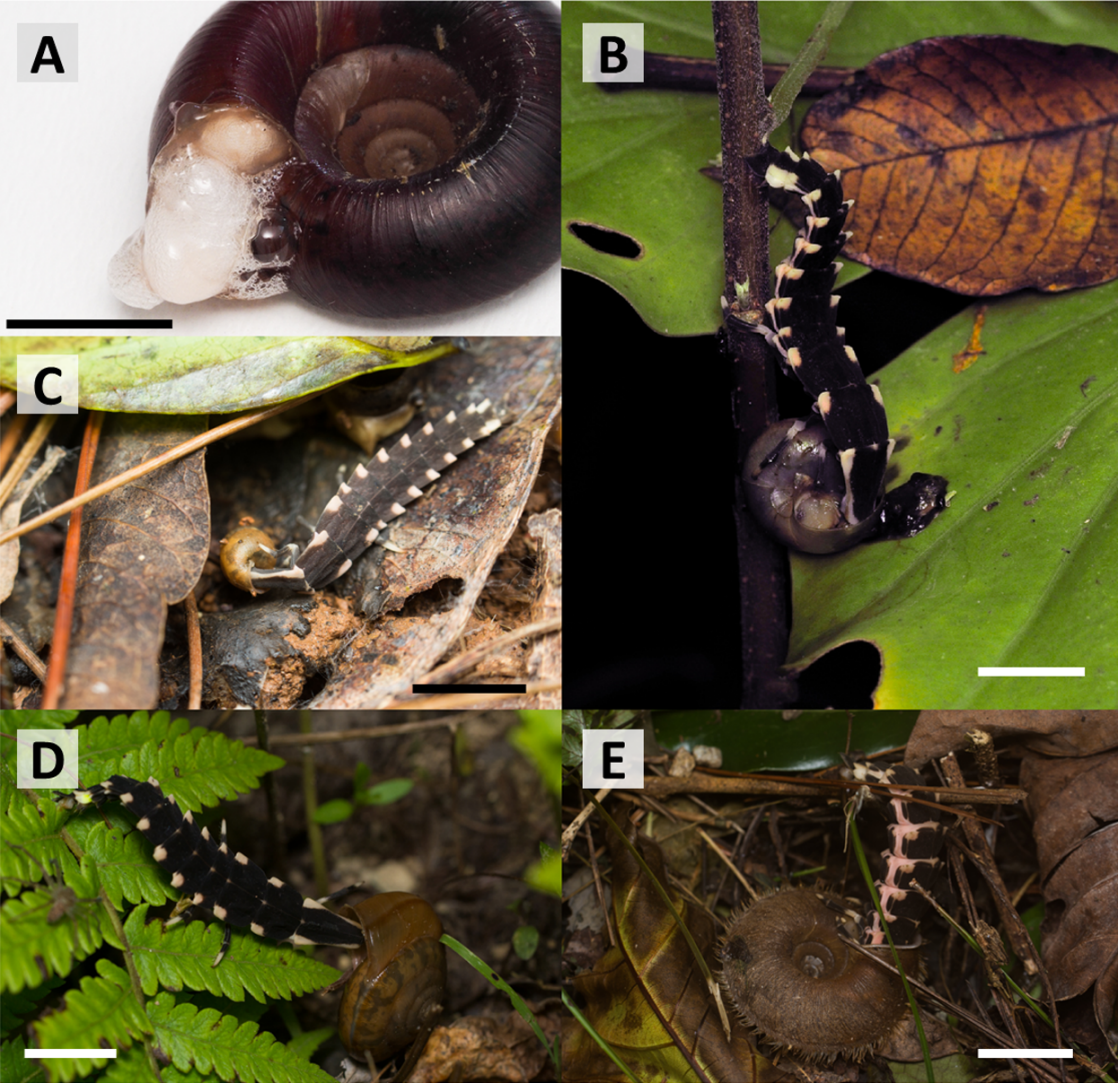
Predatory behavior of *Pyrocoelia atripennis* larvae in nature. (A) The “foamy-lid” of *Aegista vermis*, observed after a predation trial. This species fills its shell aperture with foam. (B) Last (6th) instar larva feeding on *Acusta despecta despecta* on a plant stem (July 2017, Ishigaki Island). (C) Larva feeding on *Buliminopsis meiacoshimensis* after falling to the ground following capture on a tree (October 2018, Ishigaki Island). (D) Last instar larva feeding on *Satsuma yaeyamaensis* (April 2015, Iriomote Island). (E) Last instar larva feeding on *Aegista mackensii* (October 2018, Ishigaki Island). White and black bars indicate 10-mm (photos by the author).

*P. atripennis* larvae preyed on 5 of 10 land snail species (Table 1; Table S2). Due to the operculum defense, lampyrid larvae could not prey on any Cyclophoridae species (Movies S2 and S3). Although the larvae clung to the snail searching for a gap between the operculum and shell, they usually gave up after a few min. They did not break the shell using their mandible. After failing to prey on land snails, they left. Even when they approached the snails again, they did not try to feed and appeared to have lost interest in their prey. The larvae were also unable to prey on *A. mackensii* and *A. vermis*. These ground-dwelling species (without operculum) filled their shell aperture with foam when they were attacked by lampyrid larvae (Movie S4; Fig. 4A). As this foam was discharged, the snails simultaneously drew their bodies back into the shell. In contrast, arboreal and semi-arboreal snails without an operculum, such as *Buliminopsis meiacoshimensis, A. osbeckii*, *A. d. despecta*, *S. yaeyamaensis*, *S. c. caliginosa* were preyed upon by the larvae (Movie S5). These snails are often preyed upon by *P. atripennis* larvae on plants and on the ground in nature (Figs. 4B–4D).

**Table 1:** Predation success by *P. atripennis* larvae on 10 species of land snails. A total of 21 larvae were used in this trial.

Habitat type	Land snail species	*n*	Shell width Mean ± SD (mm)	Weight Mean ± SD (g)	Operculum	Foamy-lid	Larval predation success (%)
Ground-dwelling	*Aegista mackensii*	4	32.5 ± 6.5	5.01 ± 2.07	Absent	Present	0
	*Aegista vermis*	5	24.2 ± 5.3	2.59 ± 1.10	Absent	Present	0
	*Cyclophorus turgidus radians*	3	17.4 ± 4.3	1.69 ± 1.01	Present	Absent	0
	*Cyclotus taivanus peraffinis*	3	13.0 ± 1.0	0.43 ± 0.16	Present	Absent	0
Semi-arboreal	*Acusta despecta despecta*	9	11.9 ± 2.3	0.60 ± 0.38	Absent	Absent	89
	*Satsuma caliginosa caliginosa*	4	30.0 ± 5.1	6.04 ± 3.21	Absent	Absent	25
	*Satsuma yaeyamensis*	5	22.8 ± 2.2	2.47 ± 0.77	Absent	Absent	40
Arboreal	*Aegista osbeckii*	5	11.4 ± 1.0	0.22 ± 0.06	Absent	Absent	80
	*Buliminopsis meiacoshimensis*	3	3.8 ± 0.3	0.06 ± 0.01	Absent	Absent	100
	*Leptopoma nitidum*	3	13.0 ± 1.0	0.43 ± 0.16	Present	Absent	0

## Discussion

*P. atripennis* larvae tended to choose and stay to longer on the DW trail rather than the no-trail. This may indicate kinesis to water. The mucous trail was clearly selected over no-trail and DW, and it had a long staying time which would allow the larva to carefully explore the surroundings using olfaction. This indicates not only the response to water but also recognition and tracking of the presence of prey from some components contained in land snail mucus. *P. atripennis* larvae stopped when they reached the mucous trail and moved their antennae frequently to detect the mucus. After that, they walked along the mucous trail, moving their head from side to side, and searched the surroundings. These characteristic responses following contact with mucus (defined as a “trail detection response”) and subsequent mucus tracking are commonly observed in experiments with other snail-killing predators (Mc Donnell, Paine & Gormally, 2007; Schüpbach & Baur, 2008; Iwai, Sugiura & Chiba, 2010). In the lampyrid beetles, these behaviors have already been reported by Schwalb (1961) in *Lampyris noctiluca*. Therefore, this study presents the second evidence of mucus-tracking behavior in Lampyridae and is the first record in the genus *Pyrocoelia*.

Prey preference experiments indicate that mucus of arboreal and semi-arboreal species tended to be chosen more than that of ground-dwelling species by the *P. atripennis* larvae, although larval staying time did not show a consistent trend. These results indicate that *P. attripennis* larvae can identify land snail species by their mucous trails. It also suggests that the larvae prefer the mucus of land snails that are active on plants over that of ground-dwelling species, although the results are not conclusive because of the limited number of species from each group and the limited statistical support. This tendency for predators to prefer tree snails also occurs to be in invasive flatworms and wolf snails. Sugiura & Yamaura (2010) investigated the prey-tracking ability of *Platydemus manokwari* under field conditions. They compared the survival rate of snail placed on a tree with and without mucus on the tree trunk and found that the mucous treatment resulted in more frequent predation by flatworms. This result indicates that flatworms can locate a snail on a tree using the mucous trail remaining in the trunk. And also, these snail-killing predators (including lampyrids) may detect some species-specific chemical cues from the mucus to find their favorite food.

*Euglandina rosea* prefers a smaller endemic tree snail than other terrestrial snails (Holland et al., 2012). The width of the snail’s mucus trail (volume of cues) could affect larval choice. The trails on the left and right plates were not perfectly equal because the sizes of the snail species used in Experiment 2 were somewhat different. However, shell size and the amount of mucus are not always important factors, as the smallest land snail *L. nitidum* was chosen more often by lampyrid larvae than was *A. mackensii*. Future studies should examine the cues that enable predators to recognize prey snail species separately and use more species to evaluate the generality of preference for arboreal and semi-arboreal species.

For snails, the ability to close the shell aperture is important for anti-predator defense as indicated by the observation that no snail species with an operculum was predated by lampyrid larvae in this study. The operculum defense is common in both marine and terrestrial gastropods and plays a role in avoiding predation by various predators attacking through the shell aperture (Vermeij, 2015). Above all, the operculum of the Cyclophoridae is known to have a powerful defense function that protects the soft body from very dry environments and even the digestive fluids of predators (Kelly & Cory, 1987; Norton, 1988). As a rare example, an endemic lampyrid species in Cuba, *Alecton discoidalis*, has been observed to prey on some Cyclophoridae snails under both laboratory and field conditions (Madruga Rios & Hernández Quinta, 2010). This species has a strategy that surmounts the operculum defense by waiting until the prey snail emerges again (Clench & Jacobson, 1968). However, *Pyrocoelia* larvae do not have such persistence, and they leave soon after an unsuccessful attack. Thus, keeping the operculum closed for a period of time should contribute to the survival of these snails.

Another robust defense was found in two species of the ground-dwelling snails, *A. mackensii* and *A. vermis*. These species completely blocked the larval attack by filling their shell aperture with a lump of foam. The foam produced by *Aegista* snails is very fine and sticky compared to the bubbles produced by other species (e.g., *Helicina aspersa* or *Acusta despecta despecta*) that provide only a poor defense against fireflies (Madruga Rios & Hernández Quinta, 2010; see Movie S5 in this paper). The “foamy-lid” sticks to the predator’s head, preventing invasion into the shell and functions as an anti-predatory defense as effectively as the operculum (Movie S4). To the best of my knowledge, this study is the first to report a foam defense in land snails that is highly effective against predation by fireflies. Although predation failed in all larvae in the trials, it may be successful under certain conditions. This is because *P. atripennis* larvae are sometimes observed to prey on *A. mackensii* in nature (Fig. 4E). Presumably, larval predation may be successful when snail’s defense foam cannot be produced (e.g., land snails that are weak or starving during a dry period). However, as in the present study, predation attempts will often fail because larvae cannot break through the foamy-lid of *Aegista* snails.

Arboreal snails other than *L. nitidum* can be easily preyed upon compared with ground-dwelling species, but their shells are small (the maximum diameter is approximately 15 mm), and the shell apertures are narrow. These snails are useful as prey when the larvae are young; however, they may not be desirable prey for large larvae compared to large semi-arboreal snails. The differences in the defense traits of snails may explain why larvae expend energy and time to climb trees at night, even though many species of land snail inhabit the ground surface. In the Yaeyama Islands, land snails living on the ground are exposed to many ground-dwelling predators (invasive rats *Rattus rattus* and *Rattus norvegicus*, land crabs Gecarcinidae and Potamiscinae, land hermit crabs *Coenobita*, land flatworms Bipaliidae, and some other invertebrates including other Lampyridae species); therefore, for *P. atripennis* larvae, foraging on the ground may involve substantial competition with these predators. On the other hand, semi-arboreal land snails climb plants at night when predators are active, and rest on the ground during the daytime. Although this behavior is thought to be a response to food, temperature, and humidity at night (Jaremovic & Rollo, 1979; Kasigwa, 1999), it also leads to avoidance of nocturnal ground-dwelling predators that do not climb trees. Saeki et al. (2017) reported the possibility that arboreality and seasonal vertical migration olfaction snails have adaptively evolved for the avoidance of ground-dwelling predators such as the raccoon dog (*Nyctereutes procyonide)* and carabid beetles (genus *Damaster*). These ground predators can rarely reach the arboreal prey, whereas these prey are available to the *Pyrocoelia* firefly. Tree climbing and prey-tracking in *Pyrocoelia* larvae are behaviors likely to be adapted for eating semi-arboreal or arboreal land snails without shell lids and for avoiding competitors. Moreover, the pathway of mucus that remains on the stem of the plant when semi-arboreal snails climb onto the plants from the ground surface would be expected to help the semi-arboreal lampyrid to find their prey on the plant more efficiently.

## Conclusions

This article indicates that *P. atripennis* larvae are able to track mucous trails and that they climb trees because they tend to prefer arboreal and semi-arboreal snails as their prey. Moreover, the author showed the function of the land snail operculum as a defense against firefly larvae and found a unique defense behavior consisting of a “foamy-lid” in *Aegista* snails. In the family of Lampyridae, fireflies that climb on trees and prey on land snails can be found in many genera other than *Pyrocoelia* (Wang et al., 2007) and *Lampiris* (Schwalb, 1961; De Cock, 2009). It is possible that strong selective pressure is exerted on the semi-arboreal land snails that appear to be the main foods of these tree climbing fireflies. Therefore, a future study focusing on interspecific interactions (especially predatory and defensive behaviors) between these semi-arboreal fireflies and land snails would be useful to elucidate the broader evolutionary and ecological implications of arboreality in prey-predator relationships.

## Supplemental Information

10.7717/peerj.8080/supp-1 Supplemental Information 1 RAW data of laboratory experiments.

10.7717/peerj.8080/supp-2 Supplemental Information 2 Movie S1: Experiment of mucus trail-tracking. The right plate has a “mucus trail” and the left plate has a “no-trail.”

10.7717/peerj.8080/supp-3 Supplemental Information 3 Movie S2: Predation trial of *Pyrocoelia atripennis* vs. *Cyclophorus turgidus radians*. The snail’s operculum prevents *P. atripennis* larva from entering the shell aperture.

10.7717/peerj.8080/supp-4 Supplemental Information 4 Movie S3: Predation trial of *Pyrocoelia atripennis* vs. *Leptopoma nitidum*. The snail’s operculum prevents *P. atripennis* larva from entering the shell aperture.

10.7717/peerj.8080/supp-5 Supplemental Information 5 Movie S4: Predation trial of *Pyrocoelia atripennis* vs. *Aegista vermis*. *P. atripennis* larva could not prey on this species because of the foamy-lid of *A. vermis*.

10.7717/peerj.8080/supp-6 Supplemental Information 6 Predation trial of *Pyrocoelia atripennis* vs. *Acusta despecta despecta*. This semi-arboreal snail counters predator by swinging their shell. The larva of *P. atripennis* was attached to the shell with a sucker organ, so they were not shaken off and succeeded in preying snails.

## Additional Information and Declarations

### Data Availability

The following information was supplied regarding data availability: The raw data is available in Table S1.
