# Supplementary material for: Prey-tracking behavior and prey preferences in a tree-climbing firefly
Source: PeerJ. 2019 Dec 16;7:e8080. doi: 10.7717/peerj.8080 (PMC6921978; doi:10.7717/peerj.8080)
Supplement: Supplemental Information 1 [file peerj-07-8080-s001.docx]

| Experiment  Table S1: RAW data of mucus trail-tracking (Experiment 1) and prey preference (Experiment 2). | *Pyrocoelia atripennis* larvae | | |  | Larval staying time (s) | | Larval choice |
| --- | --- | --- | --- | --- | --- | --- | --- |
|  | ID | Length (mm) | Weight (g) |  | Plate 1 | Plate 2 |  |
| Exp.1-1  Distilled water (Plate 1) vs. No trail (Plate2) | | | | | | | |
|  | A1 | 22.7 | 0.055 |  | 189 | 115 | No trail |
|  | A2 | 25.8 | 0.093 |  | 121 | 49 | Water |
|  | A3 | 24.6 | 0.064 |  | 0 | 73 | No trail |
|  | A4 | 32.2 | 0.200 |  | 71 | 88 | Water |
|  | A5 | 48.6 | 0.550 |  | - | - | Water (dropped) |
|  | A6 | 26.8 | 0.083 |  | 60 | 39 | Water |
|  | A7 | 22.5 | 0.049 |  | 41 | 54 | Water |
|  | A8 | 27.9 | 0.074 |  | 116 | 37 | No trail |
|  | A9 | 26.9 | 0.119 |  | 61 | 72 | Water |
|  | A10 | 26.4 | 0.094 |  | 310 | 117 | Water |
|  | A11 | 20.2 | 0.038 |  | 28 | 46 | Water |
|  | A12 | 43.2 | 0.420 |  | 70 | 46 | Water |
|  | A13 | 34.2 | 0.202 |  | 113 | 48 | Water |
|  | A14 | 18.0 | 0.031 |  | 87 | 143 | Water |
|  | A15 | 26.1 | 0.101 |  | 73 | 17 | No trail |
| Exp.1-2  Mucus trail (Plate 1) vs. No trail (Plate 2) | | | | | | | |
|  | B1 | 27.2 | 0.101 |  | 83 | 36 | Mucus |
|  | B2 | 25.4 | 0.077 |  | 117 | 3 | Mucus |
|  | B3 | 32.9 | 0.202 |  | 56 | 66 | Mucus |
|  | B4 | 39.1 | 0.300 |  | 70 | 0 | Mucus |
|  | B5 | 45.8 | 0.505 |  | - | - | No trail (dropped) |
|  | B6 | 40.5 | 0.945 |  | 78 | 62 | Mucus |
|  | B7 | 33.8 | 0.065 |  | 308 | 46 | Mucus |
|  | B8 | 37.3 | 0.505 |  | 72 | 55 | Mucus |
|  | B9 | 23.5 | 0.198 |  | 72 | 0 | Mucus |
|  | B10 | 49.8 | 0.253 |  | 49 | 14 | Mucus |
|  | B11 | 25.8 | 0.071 |  | 103 | 44 | Mucus |
|  | B12 | 26.8 | 0.633 |  | 84 | 0 | Mucus |
|  | B13 | 25.8 | 0.098 |  | 53 | 59 | No trail |
|  | B14 | 26.8 | 0.099 |  | 85 | 49 | Mucus |
|  | B15 | 45.2 | 0.757 |  | 97 | 46 | Mucus |
| Exp.1-3  Mucus trail (Plate 1) vs. Distilled water (Plate 2) | | | | | | | |
|  | C1 | 27.5 | 0.077 |  | 100 | 40 | Mucus |
|  | C2 | 28.8 | 0.108 |  | 35 | 58 | Mucus |
|  | C3 | 32.9 | 0.202 |  | 183 | 30 | Mucus |
|  | C4 | 19.9. | 0.036 |  | - | - | Water (dropped) |
|  | C5 | 23.5 | 0.066 |  | - | - | Mucus (dropped) |
|  | C6 | 27.1 | 0.061 |  | 123 | 46 | Water |
|  | C7 | 23.8 | 0.050 |  | 98 | 12 | Mucus |
|  | C8 | 23.9 | 0.072 |  | 114 | 24 | Mucus |
|  | C9 | 25.2 | 0.069 |  | 128 | 0 | Mucus |
|  | C10 | 43.2 | 0.494 |  | 150 | 0 | Mucus |
|  | C11 | 37.4 | 0.300 |  | 299 | 77 | Mucus |
|  | C12 | 23.3 | 0.066 |  | 122 | 91 | Mucus |
|  | C13 | 28.6 | 0.116 |  | - | - | Mucus (dropped) |
|  | C14 | 21.0 | 0.049 |  | 76 | 99 | Mucus |
|  | C15 | 30.8 | 0.196 |  | 44 | 39 | Mucus |
| Exp.2-1  *Aegista* *mackensii* (Plate 1) vs. *Leptopoma nitidum* (Plate 2) | | | | | | | |
|  | D1 | 33.5 | 0.177 |  | 44 | 54 | *Leptopoma* |
|  | D2 | 21.1 | 0.049 |  | 0 | 134 | *Leptopoma* |
|  | D3 | 20.6 | 0.038 |  | 63 | 70 | *Aegista* |
|  | D4 | 26.4 | 0.082 |  | 59 | 116 | *Leptopoma* |
|  | D5 | 24.7 | 0.061 |  | 75 | 235 | *Aegista* |
|  | D6 | 28.5 | 0.107 |  | 132 | 225 | *Leptopoma* |
|  | D7 | 24.4 | 0.085 |  | 136 | 163 | *Leptopoma* |
|  | D8 | 19.1 | 0.023 |  | 112 | 218 | *Leptopoma* |
|  | D9 | 29.5 | 0.089 |  | 100 | 60 | *Leptopoma* |
|  | D10 | 23.0 | 0.065 |  | 294 | 88 | *Aegista* |
|  | D11 | 36.1 | 0.240 |  | 79 | 115 | *Aegista* |
|  | D12 | 52.2 | 0.951 |  | 96 | 114 | *Leptopoma* |
|  | D13 | 23.6 | 0.064 |  | 72 | 72 | *Leptopoma* |
|  | D14 | 24.4 | 0.060 |  | 113 | 112 | *Leptopoma* |
|  | D15 | 26.3 | 0.087 |  | 88 | 141 | *Leptopoma* |
| Exp.2-2  *Satsuma caliginosa* (Plate 1) vs. *Leptopoma nitidum* (Plate 2) | | | | | | | |
|  | E1 | 30.2 | 0.029 |  | 307 | 36 | *Satsuma* |
|  | E2 | 19.4 | 0.077 |  | 132 | 89 | *Satsuma* |
|  | E3 | 37.6 | 0.396 |  | 124 | 41 | *Satsuma* |
|  | E4 | 45.0 | 0.457 |  | 49 | 67 | *Satsuma* |
|  | E5 | 27.5 | 0.101 |  | 116 | 63 | *Leptopoma* |
|  | E6 | 25.1 | 0.051 |  | 222 | 143 | *Leptopoma* |
|  | E7 | 32.2 | 0.147 |  | 161 | 79 | *Satsuma* |
|  | E8 | 38.2 | 0.241 |  | 554 | 202 | *Satsuma* |
|  | E9 | 25.1 | 0.070 |  | 87 | 91 | *Leptopoma* |
|  | E10 | 21.1 | 0.042 |  | - | - | *Satsuma* (dropped) |
|  | E11 | 25.0 | 0.091 |  | 181 | 100 | *Satsuma* |
|  | E12 | 26.1 | 0.083 |  | 111 | 188 | *Satsuma* |
|  | E13 | 18.7 | 0.025 |  | 218 | 134 | *Satsuma* |
|  | E14 | 42.6 | 0.052 |  | 25 | 40 | *Satsuma* |
|  | E15 | 41.6 | 0.340 |  | 120 | 141 | *Leptopoma* |
| Exp.2-3  *Sastuma caliginosa* (Plate 1) vs. *Aegista* *mackensii* (Plate 2) | | | | | | | |
|  | F1 | 18.9 | 0.033 |  | 102 | 56 | *Satsuma* |
|  | F2 | 19.2 | 0.027 |  | - | - | *Aegista* (dropped) |
|  | F3 | 27.0 | 0.082 |  | 47 | 48 | *Satsuma* |
|  | F4 | 21.5 | 0.032 |  | 309 | 74 | *Satsuma* |
|  | F5 | 32.5 | 0.184 |  | 52 | 43 | *Satsuma* |
|  | F6 | 34.4 | 0.177 |  | 182 | 158 | *Satsuma* |
|  | F7 | 45.0 | 0.457 |  | 172 | 131 | *Satsuma* |
|  | F8 | 50.2 | 0.881 |  | 103 | 361 | *Aegista* |
|  | F9 | 40..9 | 0.259 |  | 80 | 225 | *Aegista* |
|  | F10 | 23.0 | 0.093 |  | 128 | 155 | *Satsuma* |
|  | F11 | 27.8 | 0.096 |  | 346 | 189 | *Satsuma* |
|  | F12 | 25.9 | 0.071 |  | 265 | 215 | *Satsuma* |
|  | F13 | 35.0 | 0.190 |  | 29 | 43 | *Satsuma* |
|  | F14 | 30.8 | 0.137 |  | 95 | 110 | *Satsuma* |
|  | F15 | 27.9 | 0.109 |  | 45 | 40 | *Satsuma* |

| Predator: *Pyrocoelia atripennis*  Table S2: RAW data of predation trials. | | | | | | Prey snail species | | Shell width (mm) | | Wet weight (g) | | Result | |  |
| --- | --- | --- | --- | --- | --- | --- | --- | --- | --- | --- | --- | --- | --- | --- |
| ID | | | Body length (mm) | Wet weight (g) | |  |  |  |  |  |  |  |  |  |
| 1 | | | 20.0 | 0.05 | | *Cyclotus taivanus peraffinis* | | 11.8 | | 0.22 | | Failure | |  |
| 2 | | | 26.8 | 0.06 | |  | | 14.2 | | 0.61 | | Failure | |  |
| 3 | | | 25.8 | 0.09 | |  | | 13.1 | | 0.47 | | Failure | |  |
| 4 | | | 23.8 | 0.06 | | *Cyclophorus turgidus radians* | | 12.3 | | 0.51 | | Failure | |  |
| 5 | | | 23.9 | 0.07 | |  | | 12.4 | | 0.52 | | Failure | |  |
| 6 | | | 30.8 | 0.16 | |  | | 19.5 | | 2.00 | | Failure | |  |
| 7 | | | 39.1 | 0.39 | | *Aegista vermis* | | 24.8 | | 2.89 | | Failure | |  |
| 8 | | | 25.8 | 0.07 | |  | | 22.1 | | 2.41 | | Failure | |  |
| 9 | | | 45.0 | 0.76 | |  | | 30.5 | | 4.16 | | Failure | |  |
| 10 | | | 36.5 | 0.51 | |  | | 21.5 | | 1.73 | | Failure | |  |
| 11 | | | 41.0 | 0.44 | |  | | 30.6 | | 3.51 | | Failure | |  |
| 3 | | | 25.8 | 0.09 | | *Aegista mackensii* | | 21.7 | | 1.62 | | Failure | |  |
| 9 | | | 45.0 | 0.76 | |  | | 38.4 | | 7.03 | | Failure | |  |
| 12 | | | 52.1 | 0.77 | |  | | 36.6 | | 6.25 | | Failure | |  |
| 10 | | | 36.5 | 0.51 | |  | | 33.3 | | 5.14 | | Failure | |  |
| 3 | | | 25.8 | 0.09 | | *Leptopoma nitidum* | | 10.8 | | 0.31 | | Failure | |  |
| 13 | | | 25.8 | 0.03 | |  | | 10.5 | | 0.30 | | Failure | |  |
| 14 | | | 14.0 | 0.02 | |  | | 9.9 | | 0.22 | | Failure | |  |
| 15 | | | 19.0 | 0.03 | | *Aegista osbeckii* | | 13.3 | | 0.29 | | Success | |  |
| 16 | | | 18.0 | 0.03 | |  | | 11.4 | | 0.24 | | Failure | |  |
| 14 | | 14.0 | | | 0.02 | |  | | 11.2 | | 0.23 | | Success | |
| 1 | 20.0 | | | 0.05 | |  | | 10.3 | | 0.12 | | Success | |  |
| 13 | 25.8 | | | 0.03 | |  | | 11.0 | | 0.20 | | Success | |  |
| 17 | 40.5 | | | 0.91 | | *Satsuma caliginosa caliginosa* | | 28.4 | | 5.21 | | Success | |  |
| 18 | 43.0 | | | 0.38 | |  | | 24.6 | | 2.44 | | Failure | |  |
| 10 | 36.5 | | | 0.51 | |  | | 28.5 | | 5.29 | | Failure | |  |
| 19 | 42.0 | | | 0.39 | |  | | 38.4 | | 11.24 | | Failure | |  |
| 19 | 42.0 | | | 0.39 | | *Satsuma yaeyamaensis* | | 26.3 | | 3.80 | | Failure | |  |
| 20 | 33.8 | | | 0.07 | |  | | 24.0 | | 2.80 | | Failure | |  |
| 6 | 31.0 | | | 0.16 | |  | | 21.4 | | 2.04 | | Failure | |  |
| 21 | 42.0 | | | 0.24 | |  | | 22.5 | | 2.16 | | Success | |  |
| 3 | 25.8 | | | 0.09 | |  | | 19.9 | | 1.57 | | Success | |  |
| 18 | 14.0 | | | 0.02 | | *Buliminopsis meiacoshimensis* | | 4.1 | | 0.07 | | Success | |  |
| 16 | 18.0 | | | 0.03 | |  | | 3.9 | | 0.07 | | Success | |  |
| 15 | 19.0 | | | 0.03 | |  | | 3.4 | | 0.04 | | Success | |  |
| 13 | 25.8 | | | 0.03 | | *Acusta despecta despecta* | | 10.0 | | 0.25 | | Success | |  |
| 16 | 18.0 | | | 0.03 | |  | | 10.2 | | 0.16 | | Success | |  |
| 1 | 20.0 | | | 0.05 | |  | | 13.4 | | 0.82 | | Failure | |  |
| 18 | 14.0 | | | 0.02 | |  | | 7.5 | | 0.13 | | Success | |  |
| 8 | 25.8 | | | 0.07 | |  | | 15.2 | | 1.13 | | Success | |  |
| 12 | 52.1 | | | 0.77 | |  | | 13.0 | | 0.72 | | Success | |  |
| 20 | 33.8 | | | 0.07 | |  | | 10.6 | | 0.44 | | Success | |  |
| 4 | 23.8 | | | 0.06 | |  | | 12.3 | | 0.52 | | Success | |  |
| 7 | 39.1 | | | 0.39 | |  | | 14.6 | | 1.24 | | Success | |  |
